# Trauma Informed Parenting: Tailoring Interventions for Custodial Grandparents

**DOI:** 10.1093/geroni/igaf122.886

**Published:** 2025-12-31

**Authors:** Laura Shillingsburg, Danielle Nadorff, Maia McLin, Amara Mason

**Affiliations:** Mississippi State University, Starkville, Mississippi, United States; Mississippi State University, Starkville, Mississippi, United States; Mississippi State University, Starkville, Mississippi, United States; Mississippi State University, Starkville, Mississippi, United States

## Abstract

The rising number of custodial grandparents reflects a growing population navigating unique challenges. Grandparents often assume caregiving roles following traumatic events (e.g., parental substance use, incarceration), which may contribute to grandchildren’s behavioral or emotional difficulties. This abrupt transition, compounded by navigating legal, educational, and developmental demands, heightens stress for grandparents adapting to late-life parenting. Existing parenting interventions rarely address custodial grandparents’ distinct needs, particularly trauma-informed strategies and relational support. This mixed-methods study explored custodial grandparents’ experiences, stressors, and intervention preferences. First, grandparents shared insights into the circumstances prompting their caregiving role, its emotional impact, and ongoing challenges. Second, they will reviewed PC-Care—a trauma-informed parenting intervention emphasizing caregiver-child relationships—and evaluated its relevance, preferred delivery methods (e.g., virtual, in-person), and identified unmet support needs (e.g., legal guidance, peer networks). Data was collected via surveys and semi-structured interviews, transcribed, coded, and analyzed thematically to identify priorities such as accessibility, cultural sensitivity, and relational skill-building. Findings will inform adaptations of PC-Care and the development of tailored interventions addressing systemic barriers (e.g., school advocacy) and trauma-related parenting strategies. By centering grandparents’ voices, this study bridges gaps between evidence-based practices and intergenerational caregiving realities, aiming to reduce caregiver burden while fostering resilient grandparent-grandchild relationships. Outcomes will guide practitioners in designing flexible, trauma-responsive supports aligned with custodial grandparents’ lived experiences.

